# Muscular Dystrophy: Disease Mechanisms and Therapies

**DOI:** 10.1155/2015/456348

**Published:** 2015-08-24

**Authors:** Sachchida Nand Pandey, Akanchha Kesari, Toshifumi Yokota, Gouri Shankar Pandey

**Affiliations:** ^1^Research Center for Genetic Medicine, Children's National Medical Center, Washington, DC 20010, USA; ^2^Miami Children's Hospital, Miami, FL 33155, USA; ^3^Department of Medical Genetics, University of Alberta Faculty of Medicine and Dentistry, Edmonton, Canada T6G 2H7; ^4^Food and Drug Administration, Bethesda, MD 20993, USA

Progressive weakness and degeneration of skeletal muscles caused by genetic alterations fall into the category of muscular dystrophy. Muscular dystrophy occurs worldwide and affects all races. The overall incidence of muscular dystrophy varies among forms, as some forms are more common than others. Muscle loss and weakness are not necessarily caused by genetic alteration. Skeletal muscle inactivity, denervation, cancer-associated cachexia, and physiological responses to fasting or malnutrition cause skeletal muscle mass loss through imbalance in synthesis and breakdown of proteins. Several genes have been identified that are directly or indirectly involved in various muscle wasting. Studies performed in human and animal models have substantially contributed to our knowledge of molecular mechanism of muscle degeneration but still these findings are inadequate for developing effective therapy. Therefore, precise dissection of molecular mechanisms provides the way for the development of therapeutic interventions for muscular dystrophies as well as for skeletal muscle loss.

In this special issue, we intended to publish research and review articles on exploring molecular mechanisms and target identification for treatment of muscle diseases. This issue will give insight into cellular and molecular mechanisms, activation of signaling pathways, how activation of these pathways causes muscle dysfunction, and subsequent disease symptoms. The review article published in this special issue discusses the animal model for muscular dystrophy associated with dystroglycan (F. Sciandra et al.), followed by an article focusing on inflammation status and nutrition in Duchenne muscular dystrophy (DMD) patients (O. R. Cruz-Guzman et al.). The other three articles are related to cellular and molecular modeling of skeletal muscle loss. These articles describe recent advancement in the skeletal muscle research field as well as possibility for developing tools in therapeutic intervention. A study conducted by E. Guadagnin et al. provides new insight into the role of transforming growth factor beta 1 (TGF*β*1) in skeletal muscles. TGF*β*1 is recently shown to be a key player in skeletal muscle atrophy and endomysial fibrosis. E. Guadagnin et al. demonstrated that TGF*β*1 alone can induce Tyr705 phosphorylation of STAT3 in skeletal muscle cells, and higher pSTAT3 (Tyr705) leads to severe phenotype in transgenic TGF*β*1 mice.

O. R. Cruz-Guzman et al. have shown that chronic inflammation in patients with DMD may be related to loss of muscle function or to obesity. It is not known whether circulating proinflammatory cytokines such as, IL-6, IL-1, and TNF-*α* levels are associated with muscle function. Therefore, the purpose of their study was to evaluate whether an association exists between systemic inflammation with muscle function and nutritional status in DMD patient. The study concluded that systemic inflammation is increased in patients with better muscle function and decreases in DMD patients with poorer muscle function; nevertheless, systemic inflammation is similar among different levels of nutritional status in DMD patients.

Dystroglycan (DG) is highly expressed in skeletal muscle and served as extracellular matrix receptor. Mutations in components of the DG complex are cause of several muscular dystrophies such as mutations in one of the DG complex genes,* DAG1*, which has been recently associated with two forms of muscular dystrophy. In this special issue, Sciandra et al. published a review focused on the animal model systems, conditional DG knockout, and knock-in mice development, in order to study the DG function in skeletal muscle as well as in other tissues.

Hampered calcium signaling has been often reported in muscular dystrophies, which led to proteolysis of muscle protein induced by calcium ions. The assessment of intracellular calcium event is important for understanding the molecular mechanisms underlying muscular dystrophies. To understand the precise mechanism of calcium signaling in muscle cells, there is a need of robust cellular model. N. Smolina et al. have examined myotubes as a model of adult skeletal muscle for studying evoked calcium release. In addition, the authors also assessed the possibility of this cellular model for studying functional mutation effects through lentiviral transduction. Therefore, primary murine myotubes may serve as a useful cellular model for investigating calcium signaling.

C. Baligand et al. identified the key molecular pathways activated during muscle remodeling after spinal cord injury (SCI) and locomotor training in a rat model since molecular events associated with changes in muscle mass after SCI are not known completely. In this study the authors have performed genome wide expression profiling of soleus muscles at multiple time points after SCI in the well-characterize rat model. Their expression data suggest the involvement of TGF-beta/smad3 signaling in association with decrease in muscle mass observed with SCI, while the BMP pathway was activated during treadmill training. This study may provide insight into effects of BMP signaling activation and TGF*β* signaling on muscle regeneration with treadmill training in SCI through Smad3 downregulation, providing early indicators of efficient reloading in SCI model.

Articles published in this special issue will provide new insights into understanding the pathophysiology and novel therapeutic target identification of skeletal muscle diseases. As the understanding of skeletal muscle diseases improves with time, new findings will further enrich the current knowledge of these diseases, ultimately helping to develop effective therapy.

